# Correlation of symptom clusters of schizophrenia with absolute powers of main frequency bands in quantitative EEG

**DOI:** 10.1186/1744-9081-2-23

**Published:** 2006-06-29

**Authors:** Andres Gross, Sirkka-Liisa Joutsiniemi, Ranan Rimon, Björn Appelberg

**Affiliations:** 1 Helsinki University Central Hospital, Department of Psychiatry, Välskärinkatu 12 A, 00260 Helsinki, Finland/Tammiharju Hospital, 10600 Tammisaari, Finland; 2 Helsinki University of Technology, Neural Network Research Centre, 02015 Espoo, Finland./Tammiharju Hospital, 10600 Tammisaari, Finland; 3 Päijät-Häme Central Hospital, Department of Psychiatry, 15850 Lahti, Finland

## Abstract

**Background:**

Research of QEEG activity power spectra has shown intriguing results in patients with schizophrenia. Different symptom clusters have been correlated to QEEG frequency bands. The findings have been to some extent inconsistent. Replication of the findings of previous research is thus an important task. In the current study we investigated the correlations between the absolute powers of delta, theta, alpha, and beta frequency bands over the fronto-central scalp area (FC) with the PANSS subscales and the Liddle's factors in 16 patients with schizophrenia.

The authors hypothesised *a priori *the correlations reported by Harris et al (1999) of PANSS negative subscale with delta power, Liddle's psychomotor poverty with delta and beta powers, disorganisation with delta power and reality distortion with alpha power on the midline FC.

**Methods:**

The sample consisted of 16 patients with chronic schizophrenia considered as having insufficient clinical response to conventional antipsychotic treatment and evidencing a relapse. The correlations between quantitative electroencephalography (QEEG) absolute powers of delta (1.5–3.0 Hz), theta (3.0–7.5 Hz), alpha (7.5–12.5 Hz), and beta (12.5–20.0 Hz) frequency bands over the fronto-central scalp area (FC) with PANSS subscales and Liddle's factors (reality distortion, disorganisation, psychomotor poverty) were investigated.

**Results:**

Significant positive correlations were found between the beta and psychomotor poverty (p < 0.05). Trends towards positive correlations (p < 0.1) were observed between delta and PANSS negative subscale and psychomotor poverty. Alpha did not correlate with reality distortion and delta did not correlate with disorganisation.

Post hoc analysis revealed correlations of the same magnitude between beta and psychopathology generally over FC.

**Conclusion:**

The *a priori *hypothesis was partly supported by the correlation of the beta and psychomotor poverty. Liddle's factors showed correlations of the same magnitude with PANSS subscales. Supplementary analysis showed beta frequency correlating non-specifically over FC with a wide range of psychiatric symptomatology in patients with schizophrenia having a relapse.

## Background

Quantitative electroencephalography (QEEG) has enabled a detailed analysis of the background electroencephalographic (EEG) activity. Although QEEG has been proven to be a sensitive imaging modality for investigating psychiatric disorders, the adoption of QEEG by psychiatrists has been slow [1] and generally not approved for diagnostic purposes of psychiatric disorders [2].

Research of QEEG activity power spectre has shown intriguing results in patients with schizophrenia. When most of the studies have reported patients with schizophrenia evidencing increased beta and slow frequency powers and reduced main alpha power, some research show no differences and even opposite results have been reported [3]. Replication of the findings of previous research is thus an important task.

Different symptom clusters have been correlated to QEEG frequency bands. Negative symptoms have been correlated to the increase of delta [4,5] and beta [6] band activities. Positive symptoms were correlated to theta and delta activities in a magneto-encephalographic study [7]. Harris et al. [8,9] reported a stronger correlation between QEEG frequency band powers and tripartite Liddle's factors [10] of psychomotor poverty, disorganisation, and reality distortion than between the negative and positive subscales of Positive and Negative Syndrome Rating Scale (PANSS) [11]. In their studies [8,9] PANSS positive symptoms were related to Liddle's disorganisation and reality distortion, whereas psychomotor poverty was related to PANSS negative subscale. Liddle's factors showed positive correlations with delta, alpha, and beta bands. PANSS negative subscale correlated with delta power. These correlations were reported in the fronto-parietal midline electrodes [8].

In the current study we investigated the correlations between the absolute powers of delta, theta, alpha, and beta frequency bands over the fronto-central scalp area (FC) with the PANSS subscales and the Liddle's factors in 16 patients with schizophrenia. The hypothesis, based on the results of Harris et al [8] of PANSS negative subscale correlating positively with delta power, Liddle's psychomotor poverty with delta and beta powers, disorganisation with delta power and reality distortion with alpha power on the midline FC was made *a priori*. We also presumed that Liddle's factors would show a better correlation with frequency power bands than PANSS subscales. In supplementary analysis we observed the correlations between all subscales of PANSS and Liddle's factors and frequency bands over FC.

## Materials and methods

### Patients and assessments

Sixteen inpatients from Tammiharju Hospital suffering from schizophrenia were recruited to the study. All patients fulfilled the Diagnostic and Statistical Manual, Revised Third Edition (DSM-III-R) of the American Psychiatric Association (1987) criteria for schizophrenia; a senior psychiatrist made the diagnoses. Patients were considered as having insufficient clinical response to conventional antipsychotic treatment and were evidencing a relapse of the illness with PANSS mean total score 103 (range: 67–137). Patients with neurological disorders and with a history of alcohol or drug abuse were excluded from the study. Patients were recruited to the study on a voluntary basis. After being given a complete description of the study, written informed consent was obtained. The study protocol was authorised by the Ethics Committee of Tammiharju Hospital.

The mean age of the patients was 32.6 (range: 22–43) years and the male/female ratio 7/9. Three patients were diagnosed with schizophrenia of the paranoid type, one of the disorganised type, one of the residual type, and eleven of the undifferentiated type. Their mean duration of illness was 10.4 (range: 1–26) years. All patients were previously treated with conventional antipsychotics for a mean of 9.4 (SD 7.3) years. Mean daily dose of antipsychotics was 386 mg (SD 241) chlorpromazine equivalents, none had received depot antipsychotics prior to the study.

Due to clinical reasons only a short antipsychotic-free period (24–72 hours) was carried out to avoid instant effects of antipsychotics to QEEG. Neither benzodiazepines nor hypnotics were allowed in the evening preceding the QEEG registration. Patients were not routinely screened for illicit drugs as the Tammiharju Hospital is situated in a very settled suburban area, had locked door policy, and inpatients were closely monitored. Illicit substance abuse in these wards was known to be extremely rare and urine drug screens were performed if there was a clinical reason to suspect any substance misuse. Patients were also requested not to change their normal smoking pattern prior to the recordings.

The correlations of the absolute powers of QEEG frequency bands over the fronto-temporal scalp area were calculated with PANSS total score, subscales for general, positive and negative symptoms, and Liddle's tripartite factors (reality distortion, disorganisation, and psychomotor poverty). Liddle's factors were derived from 14 PANSS items describing characteristic symptoms of each factor (reality distortion – P1, P3, P6; disorganisation – P2, G5, G9, G11; psychomotor poverty – N1, N2, N3, N4, N6, N7, G7). Items were chosen on the basis of PANSS item descriptions. Clinical ratings of patients were performed blind to the QEEG results. Patients were rated within 24 hours of the corresponding QEEG recording. The same evaluator (AG) carried out all clinical assessments.

### QEEG recordings

QEEG recordings were performed between 9 a.m. – 1 p.m. During QEEG recordings the subjects were awake with their eyes closed. During recordings vigilance was controlled by visual monitoring of the prominence of the alpha frequency in the posterior parts of the brain and regular verbal contacts were maintained with the subjects. The standard 10–20 electrodes placement system with 21 electrodes on the scalp and one on both earlobes were used in the recordings. The sampling rate was 200 Hz, low-pass limit 70 Hz, and time constant (RC) 0.3 second. The digital EEG recordings of duration 10–20 minutes were saved on optical discs.

The topographical quantitative calculations were made using the Cadwell Spectrum P32 Neurometrics program, with the linked ears as a reference. The EEG digital recordings were screened visually and 48 2.5-second artefact-free epochs from the middle part of the recordings were selected from the background of the awake subject for subsequent analysis. As a result of the Fast Fourier Transformation, the averaged power spectral values of the delta (1.5–3.0 Hz), theta (3.0–7.5 Hz), alpha (7.5–12.5 Hz), and beta (12.5–20.0 Hz) bands were produced for each of the 21 scalp electrodes separately.

FC was chosen to observe on the basis of our previous study [12] where a better statistical value of correlation after averaging of electrodes on fronto-central left (FCleft) (Fp1, F3, C3), midline (FCz) (FpZ, FZ, CZ), and right (FCright) (Fp2, F4, C4) scalp areas was shown. The averaged absolute powers for FCleft, FCz and FCright were calculated for each patient separately and correlations with clinical parameters calculated.

### Statistics

Spearman's correlations were used for calculating correlations between QEEG powers and clinical parameters (PANSS subscales and Liddle's factors). An alpha level 0.05 was used as a level of statistical significance for correlation analysis of the *a priori *hypothesis.

Multivariate analysis MGLH (Multivariate General Linear Hypothesis) and General Linear Modelling, with stepwise exclusion of dependent variables, were used to test the influence of age, sex, and anxiolytic medication (diazepam equivalents) on the *a priori *hypothesized correlation.

## Results

Descriptive statistics of clinical assessments are shown in Table 1.

**Table 1:** Descriptive statistics of clinical parameters.

	P.POS	P.NEG	P.GEN	P.TOT	REAL.DIS.	DISORG.	PSY.POV.
MEAN	19.3	30.3	53.6	103.1	10.1	13.9	29.9
ST.DEV	5.8	8.1	11.4	21.6	4.3	4.2	8.1

P.POS/NEG/GEN/TOT – PANSS positive/negative/general/total subscales

REAL.DIS. – Liddle's reality distortion factor

DISORG. – Liddle's disorganisation factor

PSY.POV. – Liddle's psychomotor poverty factor

MEAN – mean value

ST.DEV – standard deviation

Correlations between absolute power values of the main frequencies on three FC areas (left, midline, and right) and PANSS scores, and Liddle's factors are shown in Table 2, the *a priori *hypothesized correlations are highlighted.

**Table 2:** Correlations between absolute powers of main frequency bands and clinical parameters.

	P.POS	P.NEG	P.GEN	P.TOT	REAL.DIS.	DISORG.	PSY.POV.
FCLEFT(B)	*0.51	a0.46	*0.60	**0.66	a0.49	**0.66	a0.47
**FCZ(B)**	0.41	a0.49	a0.46	*0.55	a0.48	*0.50	*0.52
FCRIGHT(B)	0.40	*0.52	a0.50	*0.57	a0.49	*0.58	*0.53
FCLEFT(A)	0.24	-0.04	-0.03	0.05	0.27	0.06	-0.07
**FCZ(A)**	0.23	-0.10	-0.07	0.00	**0.30**	-0.01	-0.14
FCRIGHT(A)	0.25	-0.04	-0.02	0.05	0.31	0.09	-0.08
FCLEFT(T)	0.25	0.17	0.09	0.18	0.25	0.21	0.16
FCZ(T)	a0.48	0.16	0.26	0.32	0.41	0.33	0.12
FCRIGHT(T)	0.43	0.26	0.26	0.32	a0.49	0.36	0.24
FCLEFT(D)	0.16	0.35	0.11	0.23	0.25	0.21	0.34
**FCZ(D)**	0.28	**a0.49**	0.28	0.38	a0.48	**0.34**	**a0.45**
FCRIGHT(D)	0.19	*0.52	0.18	0.32	0.36	0.30	a0.49

P.POS/NEG/GEN/TOT – PANSS positive/negative/general/total subscales

REAL.DIS. – Liddle's reality distortion factor

DISORG. – Liddle's disorganisation factor

PSY.POV. – Liddle's psychomotor poverty factor

**Bold font **indicates the correlations explaining *a priori *hypothesis

FCLEFT/Z/RIGHT – Fronto-central absolute power values of main frequencies

(D)/(T)/(A)/(B) – delta (1.5–3 Hz), theta (3–7.5 Hz), alpha (7.5–12.5 Hz), beta (12.5–20 Hz)

* – p < 0,05

** – p < 0,01

a – p < 0,1

A statistically significant (p < 0,05) positive correlation between beta power on the midline FC and psychomotor poverty was found, supporting the *a priori *hypothesis. Delta power on the FCz showed only a trend towards correlation (p < 0,1) with PANSS negative subscale and Liddle's psychomotor poverty but there was no correlation with disorganisation factors observed. Alpha power did not correlate with reality distortion factor contrary to the *a priori *hypothesis.

Positive correlations of the same magnitude, although not part of the *a priori *hypothesis were observed between beta power of three FC areas and PANSS subscales as well as Liddle's factors. Strongest correlations (p < 0.01) were seen on the FCleft between beta power and PANSS total score and Liddle's disorganisation factor (Table 2).

On the FCright the delta power showed positive correlation with PANSS negative subscale (p < 0.05).

There was no correlation between alpha and theta frequency bands and the subscales observed.

MGLH testing for the influence of sex, age, and anxiolytic medication on the *a priori *hypothesized correlation between beta power and psychomotor poverty on FCz revealed that age as a factor strengthened the correlations, other variables were rejected by the model: R^2 ^0,828; p < 0,000.

## Discussion

In patients with schizophrenia the QEEG findings on the FCz and the correlation of these findings with the clinical parameters of schizophrenia have been reported by many researchers [8,12,13].

The *a priori *hypothesis of this study was postulated on the basis of the results of Harris et al [8]. The finding of our study only partly supported the *a priori *hypothesis: the FCz significant correlation was found between beta and psychomotor poverty. The delta power showed trends towards positive correlation with PANSS negative scale and Liddle's psychomotor poverty. We did not, however, observe correlation between delta and disorganisation and between alpha and reality distortion.

The findings of Harris et al. [8], also suggested that the Liddle's tripartite factors correlated better with QEEG power spectre than the PANSS positive and negative subscales. In our study both Liddle's factors and PANSS subscales showed correlations of the same magnitude (Table 2).

Interestingly, in supplementary analysis the correlations of the same magnitude, despite being *post hoc *findings, were seen between beta power and all clinical parameters over FC (Table 2). The EEG of the FC reflects simultaneously the spontaneous function of multiple cortical areas in resting state and in patients with schizophrenia the resting cortical activity is probably altered. Psychiatric symptoms are most likely a consequence of dysfunction of multiple cortical areas and sub-cortical brain structures in patients with schizophrenia. Gaser et al. [14] indicate a pattern of distributed structural abnormalities locating in the left temporal and also right prefrontal areas being specific for auditory hallucinations. The finding supports the hypothesis of multiple cortical areas being simultaneously involved in the development of symptoms in patients with schizophrenia [14]. Based on that assumption one would expect widely distributed findings in resting EEG activity related to psychopathology in schizophrenic patients as our present findings also seem to indicate.

Beta power changes have also been consistently reported in patients with schizophrenia. One of the first observations of the EEG characteristics in patients with schizophrenia by Davis [15] was the "choppy activity" (probably consisting of a low voltage beta). It has been speculated that the increase of beta activity in schizophrenia is caused by chronic hyperarousal maintained by the brain stem reticulate activation system [16]. Dierks [17] reported an interesting finding of possible different source of beta activity in the deep structures of the brain in patients with schizophrenia (and also in depressed patients) compared to healthy controls. Correlation between overall clinical improvement, not linked to any symptom cluster, and change of beta power in the anterior areas in patients with schizophrenia was reported by Czobor and Volavka [18]. Saletu et al [19] reported the ubiquitous increase of beta power in patients with schizophrenia compared with healthy controls. There are numerous reports about correlation of symptom clusters with dysfunction of particular cortical areas which however mainly localise in fronto-temporal area [19-21].

As beta power correlated with Liddle's disorganisation and psychomotor poverty factors and also with PANSS total score, the finding indicate that beta activity may correlate non-specifically with a wide spectrum of psychopathology in patients suffering from schizophrenia as also proposed by Itil et al [22].

When testing for statistical significance of correlations between clinical parameters and QEEG measures one always runs in to the risk of making type I errors. In order to avoid that, we made the *a priori *hypothesis of PANSS negative subscale correlating positively with delta power, Liddle's psychomotor poverty with delta and beta powers, disorganisation with delta power and reality distortion with alpha power. This was based on the results of Harris et al [8], which was supported by the findings of beta power correlating with psychomotor poverty.

When it comes to the other post hoc findings of correlation between beta power and psychopathology generally the fact that similar rather strong correlations were seen on several electrodes with PANSS as well as with PANSS derived factors (Table 2), in our opinion, reduces the possibility that these are random findings. However, these findings need to be verified by future research.

Our study has a number of limitations such as a small number of subjects, a short medication-free period from antipsychotic treatment, a heterogeneous sample of patients with different length of illness and antipsychotic treatment, and no control group.

Antipsychotic medication has been shown to attenuate beta frequency power in patients responding to medication [5,22]. Our patients were, however, partial or non-responders. Furthermore, in our earlier study we showed rather minimal influence of conventional antipsychotics on the EEG power spectre [23].

Multivariate analysis was run to check the influence of benzodiazepines (BDZ) as BDZ have been shown to affect beta frequency [24]. The analysis did not evidence the BDZ dose to be a contributing factor to the correlation. The short medication-free period was determined by the clinical needs of the patients.

The question of whether the findings are illness or state dependent can not be verified with this study design. We observed the findings in the patients with schizophrenia evidencing relapse but the finding may not be limited to this patient group only.

## Conclusion

Even though the study suffers from the limitations mentioned above, some careful conclusions can be drawn from our results. The findings of Harris et al [8] regarding beta frequency showing correlation with psychomotor poverty was supported by our findings. Supplementary analysis (post hoc) suggests that the beta power non-specifically correlates on the FC with psychiatric symptomatology in patients with schizophrenia in the acute phase of the illness. This finding, however, has to be verified in future studies.

## Competing interests

The author(s) declare that they have no competing interests.

## Authors' contributions

AG participated in the designing of the study, and acquisition, analysis and interpretation of the QEEG data as well as the drafting of the manuscript. S-L J participated in acquisition and analysis of QEEG data and participated in drafting the method part of the manuscript. RR has been involved in interpretation of data, critically revising the manuscript and drafting the manuscript. BA participated in the designing of the study, performed the statistical analysis and participated in drafting of the manuscript. All authors read and approved the final manuscript.
